# *In vivo* fluorescent cercariae reveal the entry portals of *Cardiocephaloides longicollis* (Rudolphi, 1819) Dubois, 1982 (Strigeidae) into the gilthead seabream *Sparus aurata* L.

**DOI:** 10.1186/s13071-019-3351-9

**Published:** 2019-03-12

**Authors:** Gabrielle S. van Beest, Mar Villar-Torres, Juan Antonio Raga, Francisco Esteban Montero, Ana Born-Torrijos

**Affiliations:** 1 Cavanilles Institute for Biodiversity and Evolutionary Biology, Science Park, University of Valencia, P.O. Box 22 085, 46071 Valencia, Spain; 2 Institute of Parasitology, Biology Centre of the Academy of Sciences of the Czech Republic, Branišovská 31, 370 05 České Budějovice, Czech Republic

**Keywords:** *Cardiocephaloides longicollis*, Cercarial penetration pattern, Cercarial survival and activity, Metacercarial encystment, Digenea

## Abstract

**Background:**

Despite their complex life-cycles involving various types of hosts and free-living stages, digenean trematodes are becoming recurrent model systems. The infection and penetration strategy of the larval stages, i.e. cercariae, into the fish host is poorly understood and information regarding their entry portals is not well-known for most species. *Cardiocephaloides longicollis* (Rudolphi, 1819) Dubois, 1982 (Digenea, Strigeidae) uses the gilthead seabream (*Sparus aurata* L.), an important marine fish in Mediterranean aquaculture, as a second intermediate host, where they encyst in the brain as metacercariae. Labelling the cercariae with *in vivo* fluorescent dyes helped us to track their entry into the fish, revealing the penetration pattern that *C. longicollis* uses to infect *S. aurata*.

**Methods:**

Two different fluorescent dyes were used: carboxyfluorescein diacetate succinimidyl ester (CFSE) and Hoechst 33342 (NB). Three ascending concentrations of each dye were tested to detect any effect on labelled cercarial performance, by recording their survival for the first 5 h post-labelling (hpl) and 24 hpl, as well as their activity for 5 hpl. Labelled cercariae were used to track the penetration points into fish, and cercarial infectivity and later encystment were analysed by recording brain-encysted metacercariae in fish infected with labelled and control cercariae after 20 days of infection.

**Results:**

Although the different dye concentrations showed diverse effects on both survival and activity, intermediate doses of CFSE did not show any short-term effect on survival, permitting a brighter and longer recognition of cercariae on the host body surface. Therefore, CFSE helped to determine the penetration points of *C. longicollis* into the fish, denoting their aggregation on the head, eye and gills region, as well as on the dorsal fin and the lower side. Only CFSE-labelled cercariae showed a decreased number of encysted metacercariae when compared to control.

**Conclusions:**

Our study suggests that CFSE is an adequate labelling method for short-term *in vivo* studies, whereas NB would better suit *in vivo* studies on long-term performance. *Cardiocephaloides longicollis* cercariae seem to be attracted to areas near to the brain or those that are likely to be connected to migration routes to neuronal canals.

**Electronic supplementary material:**

The online version of this article (10.1186/s13071-019-3351-9) contains supplementary material, which is available to authorized users.

## Background

Host-parasite interactions represent a useful model for a better understanding of the evolution of animal life history traits and behaviours. Digeneans (Platyhelminthes: Trematoda) are particularly suitable for experimental studies and, as they are highly diverse and spread all over the world, they are becoming recurrent model systems [1–5]. Generally, their complex life-cycles involve multiple developmental stages and transmission events [6, 7]. In most life-cycles, the first intermediate host, usually a gastropod, harbours larval stages (sporocysts or rediae), which mature so that fully developed cercariae are released into the water. In most cases, these larvae infect a second intermediate host within a certain period of time (hours to days), usually another mollusc or a fish, where they encyst as metacercariae before being consumed by definitive hosts, usually fish, birds or mammals, in which the parasites complete their life-cycles by sexual reproduction [8].

Good timing of cercarial emergence in the target host habitat is crucial to ensuring contact with their host, due to the limited lifespan and energy reserves of cercariae needed until the infection of the second intermediate host [9, 10]. Therefore, glycogen reserves need to be optimized [11, 12]. Although some species are passively transmitted to the second intermediate host, generally cercariae actively infect the next host, whether by chance, invading the host anywhere along the entire body surface [13] or by a specific infective strategy in particular areas. For example, *Centrocestus armatus* cercariae specifically infect the host through the gills [14] and eye-fluke cercariae specifically infect the fish eyes by penetrating the gills and the skin (e.g. *Diplostomum spathaceum*, [15]). Once cercariae find their host, a complex process starts, with distinct phases of attachment and penetration. The penetration glands of cercariae play an important role in the penetration process into the host through the skin. Cercariae-host interaction activates their proteases and immunomodulatory activities, facilitating entrance into the host, as shown in *Schistosoma mansoni* [16, 17]. Nevertheless, there are no common attachment or penetration strategies among trematodes and, despite being essential to understand trematodes transmission, these processes remain unknown for most species, mostly because their study is highly difficult.

*In vivo* fluorescent dyes can be an adequate tool to study cercarial penetration patterns into fish. This method has been used with platyhelminths for several applications, including post-infection identification of trematode genetic clones [4], lipid utilization in *S. mansoni* [18], individual cestode tracking within intermediate host [19] and quantification of trematode larval infection success [20]. The dyes suggested in this study have been previously employed to reveal, in particular, attachment, distribution and development of monogenean larvae on fish (CFSE, [21, 22]), to localize protein kinase C in cercariae (CFSE, [23]) and to discriminate live and dead *S. mansoni* eggs (NB, [24]) and *S. mansoni* cercariae exposed to human serum (NB, [25]).

For this study, cercariae of *Cardiocephaloides longicollis* (Rudolphi, 1819) Dubois, 1982 (Strigeidae) were labelled, allowing their tracking on fish surfaces and thus their entry portals into them. The cercariae of this digenean parasite penetrate the skin and migrate into the fish brain, where they encyst as metacercariae [26]. The study of their transmission to the next intermediate host is particularly interesting as they infect fish farm species such as the gilthead seabream (*Sparus aurata* L.), which is one of the most important marine fish in the Mediterranean aquaculture, with prevalences of up to 53.9% [27]. Therefore, the present study aimed to investigate the penetration pattern of *C. longicollis* cercariae into fish. The use of fluorescent-labelling methods with cercariae is tested and evaluated by analysing their effect on cercarial survival and activity after 24 hours of labelling as well as on their viability to successfully infect and encyst in a fish host.

## Methods

### Parasite and host material

For obtaining *Cardiocephaloides longicollis* cercariae, the snail host *Nassarius reticulatus* was collected randomly during spring 2017 at the “Beach of the Eucalyptus” (40°37′35.0″N, 0°44′31.0″E) in Els Alfacs Lagoon (Ebro Delta, Spain). After acclimatisation to laboratory conditions, snails were screened for infections by incubating them individually in 96-well plates for 24 h at 25 °C, 12:12h light:dark cycle and 35 psu salinity, thereafter being checked for cercarial emergence under a stereomicroscope. To guarantee a sufficient quantity of emerged cercariae during experiments, uninfected snails were artificially infected: faecal samples were collected every two months for one year from an isolated, large colony of yellow-legged gull hosts *Larus michahellis* inhabiting an island (off the coast of Benidorm, Spain, Western Mediterranean, 38°30′07.6″N, 0°07′47.4″W). The faeces were checked under a stereomicroscope and the eggs were morphologically identified according to Dubois [28]. Identification was confirmed molecularly in random samples as described in Born-Torrijos et al. [29]. Faeces containing *C. longicollis* eggs were dropped into snails’ aquaria, and snails were periodically checked to detect infected specimens as previously described. Only newly emerged cercariae (all less than 2 h old), gathered from a pool of cercariae emerged from 12 different snail hosts, were used in the present study.

A total of 51 juvenile specific-pathogen-free (SPF) gilthead seabream (12.0 g and 6.7 cm standard length, on average), natural second intermediate hosts of *C. longicollis*, were supplied by a hatchery from Burriana (Castellón, Spain), and maintained in 3000 l tanks at the installations of SCSIE (Central Support Service for Experimental Research, University of Valencia) until their use in different experimental assays.

### Experimental procedure

#### Effect of two in vivo fluorescent dyes on cercarial survival and activity

**Fig. 1:**
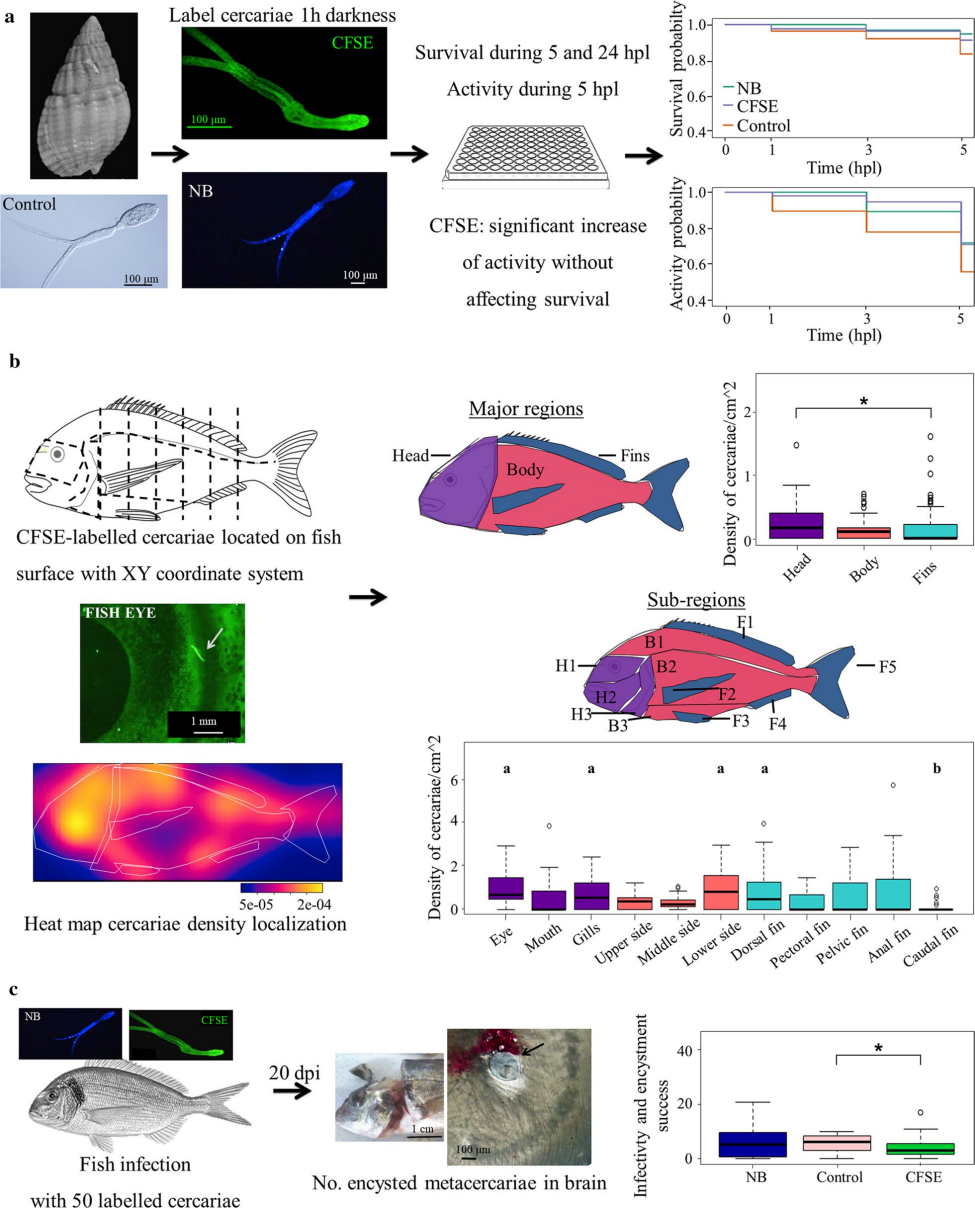
Summary of the main aims and results of the study. **a** Effect of the dye on cercarial survival and activity, recorded for 24 hours post-labelling (hpl). Survival and activity curves representing the results of the regression Weibull model (RWM) for NB and CFSE labelled cercariae within 5 hpl. **b** CFSE-labelled cercariae penetration pattern into fish observed and recorded in a XY coordinate system, divided into 3 major regions and 11 sub-regions. Major region boxplot represents the LMM results, sub-region boxplot represents pairwise comparison results, where “a” indicates the sub-regions significantly different from “b” sub-region (i.e. caudal fin, showing the lowest cercarial density). **c** Effect of the dye on cercarial infection and metacercarial encystment. The number of brain-encysted metacercariae was recorded at 20 days post-infection (dpi). The boxplot shows the GLMM results. Horizontal bars represent median values, box edges indicate interquartile range, first and third quartile, whiskers indicate the minimum and maximum values. Significant differences are marked with an asterisk or with different letters

To assess the dye effect on cercarial survival and activity, cercariae were labelled by exposing them for 60 min in dark conditions to three ascending concentrations (Fig. 1a) of two different dyes diluted with filtered seawater: (i) carboxyfluorescein diacetate succinimidyl ester (CFSE; low: 20 µM; intermediate: 50 µM; high 100 µM; Sigma-Aldrich, Saint Quentin Fallavier, France), which labels the penetration glands of the cercariae; and (ii) Hoechst 33342 (NB; low: 1 drop/ml; intermediate: 2 drops/ml; high 3 drops/ml; Thermo Fisher Scientific, Bleiswijk, Netherlands), which labels the cell DNA/nuclei. All concentrations were compared with control, unlabelled cercariae, which followed the same incubation process as labelled cercariae, but with seawater applied instead of the dye. A preliminary assay showed that parasite visualization was highest when cercariae were incubated for 60 min in dark conditions, in contrast to 10 and 30 min. After labelling, the dyeing solution was replaced with clean filtered seawater and cercariae were placed in individual 96-well plates (Fig. 1a), where they were examined under the stereomicroscope every 2 h, starting at 1 h post-labelling (hpl) for 24 h. Each concentration was tested for a total of 100 cercariae, in 4 replicates of 25 cercariae each, at room temperature (*c.*25 °C), 12:12 h light:dark cycle and 35 psu salinity. Cercarial activity was classified by visual assessment as active (swimming cercariae) or inactive (cercariae barely swimming, showing erratic movements and spontaneous spasms), while cercarial longevity was recorded as the time until cercariae did not show spasmodic movements.

#### Penetration pattern on host surface

Fish were infected with labelled cercariae and analysed to determine the parasite penetration pattern on the host body surface (Fig. 1b). Fifteen *S. aurata* were infected with 100 cercariae each. These were previously labelled with an intermediate CFSE concentration under the same conditions described above. After this process, cercariae were maximum 6 h old. Additionally, unlabelled cercariae and fish tissue were examined under a fluorescence stereomicroscope (MZZ16F; Leica, Wetzlar, Germany) to discard any natural fluorescence that could interfere with the recorded data. Immediately following fish euthanasia by concussion to avoid the detachment of cercariae by chemicals, fish were placed into a black box with enough filtered seawater to cover them (90 mm^3^) and their body surface was observed under a fluorescence stereomicroscope equipped with a digital camera (MZZ16F Leica, DFC300 FX Leica). Both fish flanks, randomly selecting the first side to be analysed, were carefully examined for live parasites for an average time of 20 min/fish flank. Parasite location was recorded on an XY coordinate system based on a grid representing a fish flank, divided into different regions (Fig. 1b). Some zones, such as mouth, eyes and gills, were considered as particular zones as they may be common penetration areas for trematodes. Gills, oral folds, oral cavity, nasal chamber and ventral area were also examined. Scaled photographs were taken of each fish and this information was transferred into a digital XY coordinate system together with the location data in the free image-processing program ImageJ v.1.47 [30].

#### Dye effect on cercarial infectivity and metacercarial encystment in brain

To assess the effect of the fluorescent dyes on cercarial infectivity and later metacercarial encystment, control and NB or CFSE labelled cercariae were used to infect fish (Fig. 1c). After the acclimatisation of 36 fish in individual cylindrical containers with enough filtered seawater to cover them (*c.*200 cm^3^), fish were exposed to 50 cercariae for 60 min (3 replicates of 4 fish for each treatment, using intermediate dye concentrations). Thereafter, complete cercariae, tails and bodies that remained in the water after fish infection were counted to have an accurate initial infection value (3.5 ± 0.3 complete cercariae and bodies were found after experiment in all treatments). The fish were measured, weighed and maintained in aquaria at room temperature, then euthanized at 20 days post-infection (dpi) with MS222 (tricaine methanesulfonate, 0.03% solution buffered in seawater; Sigma-Aldrich). The number of metacercariae in the brain was then recorded after meticulous skull dissection (Fig. 1c).

### Statistical analysis

To assess the variation in survival and activity rates of cercariae and investigate differences between concentrations in each treatment, data were tested by mixed model Cox proportional hazards regression (MMCoxPH, package *coxme*, [31]). If data did not show a constant proportional hazard between levels (validated at *P* > 0.05 with function *cox.ph*, package *survival*), a regression Weibull model (RWM) was used (package *survival*, [32]) instead of MMCoxPH. Survival analyses aim to model time to event data, usually considering death as the event. In this study, we used the death of a cercaria as the event to study their survival, and for studying cercarial activity, the inactivity of a cercaria was considered the event. First, to evaluate the effect of the concentration of each dye on the survival and activity of cercariae, survival analyses were used for each treatment separately (CFSE and NB), with concentrations as a covariate (i.e. control, low, intermediate and high) in the RWM and in the MMCoxPH model (here adding the control treatment as the baseline with which the dye treatment were compared to, and replicates as random effect). Differences were evaluated by comparing the different concentrations of each fluorescent dye to the control treatment. Survival of cercariae was analysed after 5 hpl, i.e. the time during which the penetration pattern assay was run, and after 24 hpl, whereas the activity of cercariae was analysed after 5 hpl. To explore the survival and activity of labelled cercariae after 5 hpl, differences between intermediate concentrations of both dyes were analysed. For both survival and activity models, intermediate concentration of NB and CFSE was considered as a covariate and compared to control cercariae.

To study the *C. longicollis* penetration pattern, data on both fish flanks were combined after discarding significant differences between the number of cercariae attached to the left and right body flanks by a generalized linear model fitted with a quasibinomial structure (GLM; logit link function, package *lme4*, [33]), using the proportion of attached cercariae as response variable and the fish flank as fixed effect (*P *> 0.05, Additional file 1: Table S1). In order to detect any pattern of the parasite distribution on the host’s body surface, the positions of cercariae were analysed by point process models (PPM; package *spatstat*, [34]). For these two dimensional parasite distribution analyses, parasites attached underneath the pectoral fins had to be excluded, resulting only in the omission of 2 cercariae and thus including the rest attached to both fish flanks. A complete spatial randomness simulation (CSR) was created based on Quadrat counting test and Kolmogorov-Smirnov test to confirm the assumption of uniformity of *C. longicollis* cercariae distribution on the fish surface. A heat map was created to illustrate their position, using kernel density analysis (see Fig. 1b).

To detect the entry portals into the fish, the fish surface was first divided into 3 major regions [see Fig. 1b, head (H), body (B) and fins (F)] and afterwards into 11 regions (see Fig. 1b), subdividing the head into 3 sub-regions [eye (H1), mouth (H2), gills (H3)], the body into 3 sub-regions [upper (B1), middle (B2) and lower (B3) side], and the fins into 5 sub-regions [dorsal (F1), pectoral (F2), pelvic (F3), anal (F4) and caudal (F5) fins]. To obtain the number of parasites for each region, the parasite counts were pooled from the XY coordinate grid data following the previously described regions (shown in Fig. 1b). Differences between regions were analysed by linear mixed models (LMM; package *lme4*) using Box-Cox transformed values [35] of cercariae density [number of cercariae on region / total number of cercariae on fish]/[region area (cm^2^)/total fish area (cm^2^)] as response variable, the established regions as fixed effect and the replicates as random effect.

To assess the effect of the dye on cercarial infectivity and metacercarial encystment in the fish’s brain, differences in the number of encysted metacercariae were analysed by generalized linear mixed model fitted with a binomial structure (GLMM; logit link function, package *lme4*), using the proportion of brain-encysted metacercariae as response variable, the dye type as fixed effect and replicates as random effect.

After LMM and GLMM, multiple pairwise comparisons were obtained using Tukey’s all-pair comparisons *post-hoc* test (package *multcomp*, [36]), adjusting *P*-values with Bonferroni correction. All statistical analyses were performed in R (R Development Core Team, version 3.0.1), with significance set at α = 0.050.

## Results

### Survival and activity of labelled cercariae

Our analyses showed a significant decrease in the mortality risk, i.e. 29.9, 67.9 and 57.3% longer survival of NB-labelled cercariae after 24 hpl in low, intermediate and high concentration, respectively, compared to the control [RWM low, *P* = 0.0140 (HR = 0.70, 95% CI: 0.53–0.93); intermediate, *P* < 0.0001 (HR = 0.32, 95% CI: 0.24–0.43); high, *P* < 0.0001 (HR = 0.43, 95% CI: 0.32–0.57); Additional file 2: Table S2i]. After 5 hpl, i.e. the time necessary to run the penetration assay (see Penetration pattern on host surface in Methods), both intermediate and high concentration NB-labelled cercariae showed a significant increase of survival when compared to control, when mortality risk decreased by 71.5 and 70.1%, respectively [MMCoxPH, intermediate, *P* = 0.0150 (HR = 0.28, 95% CI: 0.10–0.79); high, *P* = 0.0190 (HR = 0.30, 95% CI: 0.11–0.83); Additional file 2: Table S2ii]. At this time, cercarial activity significantly increased at all NB concentrations when compared to control cercariae, showing a reduction of the risk of inactivity (longer activity time) in low (57.7%), intermediate (55.7%) and high (62.7%) concentration [RWM, low, *P* = 0.0011 (HR = 0.42, 95% CI: 0.26–0.70); intermediate, *P* = 0.0009 (HR = 0.44, 95% CI: 0.28–0.77); high, *P* = 0.0003 (HR = 0.37, 95% CI: 0.22–0.63); Additional file 3: Table S3].

On the other hand, the survival rate of CFSE-labelled cercariae showed a significant increase, showing a reduction in the mortality risk of 46.0% at low concentrations compared to control after 24 hpl [RWM, *P* < 0.0001 (HR = 0.54, 95% CI: 0.40–0.72); Additional file 4: Table S4i], but did not show any significant differences after 5 hpl (MMCoxPH, *P* > 0.05; Additional file 4: Table S4ii). The activity rate of CFSE-labelled cercariae after 5 hpl showed a significant increase as the probability of the risk of inactivity decreased by 66.2% in low concentration and 39.8% in intermediate concentration when compared to control [RWM, low, *P* = 0.0002 (HR = 0.34, 95% CI: 0.20–0.58); intermediate, *P* = 0.0420 (HR = 0.60, 95% CI: 0.37–0.97); Additional file 5: Table S5].

The RWM analyses comparing survival and activity rates between intermediate concentrations of NB and CFSE after 5 hpl showed that NB-labelled cercariae exhibit significantly higher cercarial survival, by reducing the mortality risk by 78.4%, and the inactivity risk by 57.1% compared to control cercariae [RWM, survival, *P* = 0.0062 (HR = 0.22, 95% CI: 0.08–0.60); activity, *P* = 0.0006 (HR = 0.43,95% CI: 0.27–0.69); Additional file 6: Table S6], while CFSE labelled cercariae did not show significant differences in survival rates (*P* > 0.05; Additional file 6: Table S6i) and 39.8% significantly lower inactivity risk than control cercariae [*P* = 0.0429 (HR = 0.60, 95% CI: 0.37–0.97); Additional file 6: Table S6ii, Fig. 1a]. This, together with our preliminary visual assessment, showed that the most adequate fluorescent dye and dose was the CFSE intermediate concentration, with longer permanence and a powerful brightness that permits easy recognition of labelled cercariae for 5 h. Thus, this concentration was chosen to label cercariae used in the following experiments.

### Penetration pattern of *C. longicollis* into the fish host

Our results pointed to a non-uniform distribution of *C. longicollis* cercariae on the host’s body surface determined by CSR simulation based on Quadrat counting test and Kolmogorov-Smirnov test (*χ*^2^ = 142.66, *P* < 0.0001 and *χ*^2^ = 61.83, *P* < 0.0001, respectively; Fig. 1b). The heat map illustrates the aggregation of attached fluorescent cercariae especially close to the head, eye and gills region, as well as to the dorsal area of the fish. Furthermore, analyses where the surface was divided into 3 major regions indicated a significant higher cercarial density on the head region compared to the fins, showing 1.10 times more cercariae (LMM, *F*_(2,330)_ = 6.00, *P* = 0.0006; Additional file 7: Table S7i, Fig. 1b), this being supported by pairwise comparisons (Additional file 7: Table S7ii). When comparing cercarial density between sub-regions, our results showed that the density of cercariae was not significantly different between the eyes (H1), dorsal fin (F1), lower side (B3) and gills (H3) (LMM, *F*_(10,330)_ = 3.89, all *P* > 0.05), these areas being the most attractive ones. The first two showed significant differences with almost all other regions, while lower side and gills only showed significantly 1.57 and1.55-fold higher density than the caudal fin, respectively (LMM, both *P* = 0.0002). The eye region, which was the area showing the highest cercarial density, significantly differed from less attractive areas by a range of 1.38–1.85-fold higher (LMM, *P* = 0.0246, *P* < 0.0001, respectively; Additional file 8: Table S8i), with the caudal fin (F5) showing the lowest density (*P* < 0.0001). The dorsal fin, the second most attractive area, showed a significant different density that ranged between 1.36–1.71-fold higher (LMM, *P* = 0.0220, *P* < 0.0001, respectively), which included the mouth and the rest of fins, with the caudal fin being once again the one with the significantly lowest density (*P* < 0.0001). Pairwise comparisons only supported significant differences between the four most attractive areas (eye, dorsal fin, lower side and gills) and the caudal fin (F5) (pairwise comparisons, *P* < 0.0001, *P* = 0.0006, *P* = 0.0065, *P* = 0.0075, respectively, see Fig. 1b; Additional file 8: Table S8ii).

### Dye effect on cercarial infectivity and metacercarial encystment

When looking at the differences in cercarial infectivity after labelling, our results showed that the number of encysted metacercariae after infection was not significantly different between control and NB-labelled cercariae (GLMM, *F*_(2,36)_ = 4.17, *P* > 0.05; Additional file 9: Table S9, Fig. 1c). However, CFSE-labelled cercariae showed 1.62 times significantly less infection and later metacercarial encystment than control cercariae (GLMM, *P* = 0.0039; Additional file 9: Table S9i, Fig. 1c). Pairwise comparisons were concordant with GLMM results (Additional file 9: Table S9ii).

## Discussion

In this study, *in vivo* fluorescent labelling allowed the tracking of the distribution of *C. longicollis* cercariae during penetration and infection of the host, pointing to the eye region, gills, lower side and dorsal fin as the areas where cercariae attach most frequently. Furthermore, the investigation of the effects of two different fluorescent dyes on the survival and activity of cercariae, as well as on their viability to later infect and encyst in the target organ, permitted identification of the most adequate dose and substance to label trematode cercariae used in experiments. All cercariae labelled with NB or CFSE dyes were detectable up to 24 h and probably longer, although this was not further explored.

### Short- and long-term effects of NB and CFSE fluorescent dyes

Ascending concentrations of both dyes were tested by observing labelled cercariae for 24 hpl, i.e. longer than needed for the fulfilment of the rest of experiments. Of particular interest are the first 5 h after cercarial emergence from the snail host and their labelling, since, besides being the time needed to complete the penetration pattern experiment, it coincides with the highest infective period previously described for cercariae (e.g. 3–6 h in *D. spathaceum* [37]; 1–9 h in *S. mansoni* [38]). Thus, the study of specific effects of fluorescence during this period is crucial. Overall, NB showed a slight increase of cercarial survival in every concentration, as well as an increase of their activity after 5 hpl. The free-living cercarial stage is short-lived and non-feeding, and is dependent on glycogen storage for energy metabolism [8, 10]. Nevertheless, it is well known that external factors, including biotic and abiotic factors, may affect the lifespan of trematodes. Several publications have recently drawn our attention to toxic pollutant effects on trematode free-living stages, since they could affect cercarial survival and activity or infection success: for example, cadmium and zinc in different species, such as *D. spathaceum* [39, 40]; mercury in *Diplostomum* sp. [41]; or herbicides and pesticides in several species [42–44]. The precise mechanisms by which toxic compounds may affect cercarial behaviour remain unknown. In our study, NB showed a much larger effect on cercarial survival and activity, increasing both of them. An alteration of the neuromuscular stimulation that controls cercarial swimming (e.g. increased serotonin or decreased acetylcholine [45]) or a more efficient consumption of glycogen reserves affected by the labelling of the nucleic acid by NB could explain these effects (e.g. cytoxic and kinetic responses rely on the different cell types, even sometimes altering their viability and/or proliferative behaviour, as suggested by Fried et al. [46]). Overall, CFSE seemed to have a smaller effect on cercarial behaviour, increasing their activity during the first 5 hpl but not their survival. Although mechanisms affected by external substances should be further studied, the objective in the present study was to uncover which fluorescent dye affected short-term cercarial performance; it was demonstrated that the intermediate CFSE concentration exhibited the lowest effect on this performance. On the other hand, CFSE was the only dye that affected cercariae in the long-term since it decreased fish infection, with 1.62 times lower metacercarial encystment, showing that it affects cercariae infective capacity. CFSE labels the penetration glands of cercariae, which have an important role during the penetration into the host’s skin due to their high protease activity [16, 17]. Hence, labelling the penetration glands with CFSE could either inhibit their enzymes or lessen their stimulation, thus affecting their capacity to recognize the host. Therefore, NB dye, which did not significantly affect *C. longicollis* cercarial infective capacity, would be a more adequate dye in *in vivo* studies on long-term larval performance.

Our study supports previous studies where CFSE labelling was proven as an appropriate method for a rapid and easy *in vivo* dye for small and translucent infective stages, e.g. myxosporean actinospores [47], monogenean oncomiracidia [21], and even for experiments on specific organs such as the study of the penetration glands activation process in cercariae by Ressurreição [see 23]. Although this method has been proven to affect long-term cercarial performance by decreasing infection success and metacercarial encystment, it is adequate to study short-term survival and activity, allowing us to reveal the penetration pattern of *C. longicollis* cercariae into the fish host (see later). To our knowledge, NB has been previously used only for the discrimination between live and dead *S. mansoni* eggs [24] and cercariae after exposure to human serum [25], whereby a less cell-permeant version of the dye was used. Thus, this is the first time that survival and activity rates in unlabelled and NB-labelled cercariae have been compared. We consider NB (Hoechst 33342) as a useful tool for differentiation between dead and live larval stages, but more studies on long-term survival of labelled cercariae are necessary before using this dye in experimental assays, e.g. testing drug-resistance in human parasites.

### Penetration pattern of *C. longicollis* larvae into the fish host

Little is known about common attachment and penetration patterns of trematodes into their hosts and each trematode species probably develops a pattern with specific entry portals into the fish hosts. In this study, although *C. longicollis* cercariae showed a non-random distribution, they could be detected attached all over the fish’s surfaces, but more frequently penetrating areas as close as possible to the brain, such as the eyes, gills, dorsal fin and lower side. The rest of the fins, the mouth area and the more muscular surfaces of the body, i.e. upper and middle body side, showed slightly smaller numbers of attached cercariae, although none so few as the caudal fin, which seemed to be a much less attractive entry point into the fish. Once the cercariae contact a possible host by chance, the specificity for fish invasion is achieved by contacting carbohydrates present in the mucus covering the hosts (see [48] for *D. spathaceum* cercariae), such as happens in cercariae of *Opisthorchis viverrini*, where the attachment and penetration relies on the adequate identification of glycosaminoglycans and proteins present on the fish surface [49]. However, specific interactions between a parasite species and its host are an effective strategy to complete their life-cycle. Cercariae usually establish a suitable entry portal that can guarantee a higher infection success, such as human skin for schistosomes (e.g. *S. mansoni* and *S. haematobium*, [11]) or the gills in different trematodes (e.g. *C. armatus*, [14]). Some cercariae, like those of eye-flukes, usually penetrate through the gills and skin to infect the eye (*D. spathaceum*, [48]). Fish mucus attractants could help cercariae to trace, by chemoreception, a favourable entry portal into the fish [48], but although host-recognition mechanisms seems to be crucial for their transmission, many species reach their target only after opportunistically penetrating the host, which can risk losing cercariae in a dead end host [50].

Many strigeoids migrate great distances within the host, from the cutaneous entry point to the target organ, as for example *Ornithodiplostomum ptychocheilus*, whose metacercariae also establish in the brain of its intermediate fish host [51, 52]. However, and to the best of our knowledge, although the migrations routes are better known, the specific or preferred entry portals are still unclear for brain-encysting metacercariae. When comparing areas within the head, the eyes were highlighted as one of the most attractive areas, together with the gills, which accommodated more cercariae than the oral cavity (Additional file 10: Table S10). In any case, this study demonstrated that a high density of attached cercariae concentrate on areas which might connect and transport them faster to the brain. Therefore, for instance, the penetration of the head of the host and especially the gills may facilitate access to the blood in the circulatory system, as happens for example in *D. spathaceum* when infecting the eyes [53]. On the other hand, the concentration of cercariae on the lower side might be related to the presence of the pelvic girdles, which are close to the head region in this fish species (see Fig. 1b), and cercariae penetrating the host by the dorsal fin could use peripheral nerves to access the central nerve cord or cranial nerves to directly reach the brain, as proposed in the studies with *O. ptychocheilus* cercariae of Hendrickson & Matisz [53, 54]. Hendrickson [52] also suggested that the neuronal canal could be also easily accessed from the caudal fin, even more rapidly than cercariae penetrating in areas much closer to the brain, but this route seems not to be preferred by *C. longicollis* since the caudal fin showed fewer cercariae attached on its surface. Thus, overall, infection success of *C. longicollis* cercariae might result from the important attraction shown for areas related to the brain, either by their proximity or by the easy migration routes to reach it.

## Conclusions

This study allows for a better understanding of the cercarial transmission and penetration of *C. longicollis* into its fish host, by using optimized detection methods that facilitated our *in vivo* study on larval performance and offered a better visualization of cercariae on the fish surface. Our results validated CFSE as an adequate method to study short-term larval performance, while NB serves better for long-term studies, and also demonstrated that *C. longicollis* cercariae show an attraction to areas near to the brain or with easy access to migration routes. Future studies on possible migration routes to target organs within the fish will help elicit a better understanding of strigeoid life-cycles.

## Additional files

**Additional file 1: Table S1.** Evaluation of the effect of fish’s flank on cercarial attachment success.

**Additional file 2: Table S2.** Evaluation of the effect of different NB concentrations on cercarial survival.

**Additional file 3: Table S3.** Evaluation of the effect of NB concentration treatment on cercarial activity.

**Additional file 4: Table S4.** Evaluation of the effect of CFSE different concentrations on cercarial survival.

**Additional file 5: Table S5.** Evaluation of the effect of CFSE concentration treatment on cercarial activity.

**Additional file 6: Table S6.** Evaluation of the effect of dyes on cercarial survival and activity.

**Additional file 7: Table S7.** Evaluation of the effect of major regions of fish’s surface on cercarial density.

**Additional file 8: Table S8.** Evaluation of the effect of sub-regions of fish’s surface on cercarial density.

**Additional file 9: Table S9.** Evaluation of the effect of dyes on cercarial infectivity and metacercarial encystment success.

**Additional file 10: Table S10.** Evaluation of the effect of fish’s head sub-regions on cercarial density.

## Abbreviations

CFSE: carboxyfluorescein diacetate succinimidyl ester
CI: confidence interval
CSR: complete spatial randomness simulation
dpi: days post-infection
GLM: generalized linear model
GLMM: generalized linear mixed model
hpl: hours post-labelling
HR: hazard ratio
LMM: linear mixed models
MMCoxPH: mixed model Cox proportional hazards regression
NB: Hoechst 33342
PPM: point process models
RWM: regression Weibull model
