# Supplementary material for: In vivo fluorescent cercariae reveal the entry portals of Cardiocephaloides longicollis (Rudolphi, 1819) Dubois, 1982 (Strigeidae) into the gilthead seabream Sparus aurata L
Source: Parasit Vectors. 2019 Mar 12;12:92. doi: 10.1186/s13071-019-3351-9 (PMC6417200; doi:10.1186/s13071-019-3351-9)
Supplement: Supplementary file 1 — Additional file 1: Table S1. Evaluation of the effect of fish’s flank on cercarial attachment success. [file 13071_2019_3351_MOESM1_ESM.docx]

**Additional file 1: Table S1.** Evaluation of the effect of fish’s flank on cercarial attachment success.

No significant effect of fish’s flank on cercarial attachment success.

|  | **Estimate** | | **SE** | | ***z-value*** | | **P-value** | |
| --- | --- | --- | --- | --- | --- | --- | --- | --- |
| **GLM** | | | |  |  | | |  |
| **Intercept (=Left flank)** | | -3.0766 | 0.1157 | | | -26.5970 | **<0.0001** | |
| **Right flank** | | 0.0679 | 0.1611 | | | 0.4220 | 0.6740 | |

Results evaluating with generalized linear model (GLM) (Proportion of attached cercariae ~ Fish flank). The intercept value in the GLM stands for the mean number of cercariae successfully attached to the left fish flank on the logit scale, to which the right flank is compared. The estimate of the right flank is added to the intercept value. Statistically significant results (at α = 0.050) are indicated in bold.
