# Supplementary material for: In vivo fluorescent cercariae reveal the entry portals of Cardiocephaloides longicollis (Rudolphi, 1819) Dubois, 1982 (Strigeidae) into the gilthead seabream Sparus aurata L
Source: Parasit Vectors. 2019 Mar 12;12:92. doi: 10.1186/s13071-019-3351-9 (PMC6417200; doi:10.1186/s13071-019-3351-9)
Supplement: Supplementary file 2 — Additional file 2: Table S2. Evaluation of the effect of different NB concentrations on cercarial survival. [file 13071_2019_3351_MOESM2_ESM.docx]

**Additional file 2: Table S2**. Evaluation of the effect of different NB concentrations on cercarial survival.

Increase of survival of cercariae labelled with the three concentrations of NB dye after 24 hpl. Increase of survival in the intermediate and high concentrations after 5 hpl.

|  | **Estimate** | **SE** | ***z-value*** | **P-value** |
| --- | --- | --- | --- | --- |
| **(i) RWM** |  |  |  |  |
| **Survival 24 hpl** |  |  |  |  |
| **Intercept (=Control)** | 2.3452 | 0.0331 | 70.9400 | **<0.0001** |
| **Low Concentration** | 0.1103 | 0.0451 | 2.4500 | **0.0140** |
| **Intermediate Concentration** | 0.3517 | 0.0447 | 7.8700 | **<0.0001** |
| **High Concentration** | 0.2627 | 0.0451 | 5.8200 | **<0.0001** |
| **Log(scale)** | -1.1756 | 0.0397 | -29.6000 | **<0.0001** |
|  | **exp^(β)^** | **se(β)** | ***z-value*** | **P-value** |
| **(ii)MMCoxPH** |  |  |  |  |
| **Survival 5 hpl** |  |  |  |  |
| **Low Concentration** | 0.4709 | 0.4379 | -1.7200 | 0.0860 |
| **Intermediate Concentration** | 0.2849 | 0.5166 | -2.4300 | **0.0150** |
| **High Concentration** | 0.2989 | 0.5167 | -2.3400 | **0.0190** |

Results evaluating (i) the effect of different concentrations of NB on cercarial survival after 24 hpl with Weibull regression model (RWM) (alive cercariae ~ NB concentration) and (ii) after 5 hpl with mixed model Cox Proportional Hazards regression (MMCoxPH) (alive cercariae ~ NB concentrations + replicates (random)). The intercept in (i) stands for the survival rate of the control cercariae, to which the other three levels are compared, i.e. low, intermediate and high NB concentration. The hazard rate (exp^(β)^) of control cercariae in (ii) is 1, to which the other levels are compared. If exp^(β)^ < 1, mortality risk is reduced. Statistically significant results (at α = 0.050) are indicated in bold. We also provide the scale parameter which indicates with log(scale) the Weibull distribution estimation.
