# Supplementary material for: In vivo fluorescent cercariae reveal the entry portals of Cardiocephaloides longicollis (Rudolphi, 1819) Dubois, 1982 (Strigeidae) into the gilthead seabream Sparus aurata L
Source: Parasit Vectors. 2019 Mar 12;12:92. doi: 10.1186/s13071-019-3351-9 (PMC6417200; doi:10.1186/s13071-019-3351-9)
Supplement: Supplementary file 3 — Additional file 3: Table S3. Evaluation of the effect of NB concentration treatment on cercarial activity. [file 13071_2019_3351_MOESM3_ESM.docx]

**Additional file 3: Table S3**. Evaluation of the effect of NB concentration treatment on cercarial activity.

Increase in activity of cercariae labelled with the three concentrations of NB dye after 5 hpl.

|  | **Estimate** | **SE** | ***z-value*** | **P-value** |
| --- | --- | --- | --- | --- |
| **RWM** |  |  |  |  |
| **Activity 5 hpl** |  |  |  |  |
| **Intercept (=Control)** | 2.5256 | 0.1421 | 17.7800 | **<0.0001** |
| **Low Concentration** | 0.7456 | 0.2285 | 3.2600 | **0.0011** |
| **Intermediate Concentration** | 0.7072 | 0.2136 | 3.3100 | **0.0009** |
| **High Concentration** | 0.8565 | 0.2359 | 3.6300 | **0.0003** |
| **Log(scale)** | -0.1380 | 0.0848 | -1.6300 | 0.1037 |

Results of regression Weibull model (RWM) evaluating the effect of NB concentration treatment on cercariae activity rate after 5 hpl (active cercariae ~ NB concentration). The intercept value stands for the activity rate of the control, to which the other three levels are compared, i.e. low, intermediate and high NB concentration. Statistically significant results (at α = 0.050) are indicated in bold. We also provide the scale parameter which indicates with log(scale) the Weibull distribution estimation.
