# Supplementary material for: In vivo fluorescent cercariae reveal the entry portals of Cardiocephaloides longicollis (Rudolphi, 1819) Dubois, 1982 (Strigeidae) into the gilthead seabream Sparus aurata L
Source: Parasit Vectors. 2019 Mar 12;12:92. doi: 10.1186/s13071-019-3351-9 (PMC6417200; doi:10.1186/s13071-019-3351-9)
Supplement: Supplementary file 4 — Additional file 4: Table S4. Evaluation of the effect of CFSE different concentrations on cercarial survival. [file 13071_2019_3351_MOESM4_ESM.docx]

**Additional file 4: Table S4**. Evaluation of the effect of CFSE different concentrations on cercarial survival.

Increase of survival of cercariae labelled with low concentration of CFSE dye after 24 hpl. No significant effect of CFSE dye after 5 hpl.

|  | **Estimate** | **SE** | ***z-value*** | **P-value** |
| --- | --- | --- | --- | --- |
| **(i) RWM** |  |  |  |  |
| **Survival 24 hpl** |  |  |  |  |
| **Intercept (=Control)** | 2.3460 | 0.0330 | 71.0800 | **<0.0001** |
| **Low Concentration** | 0.1892 | 0.0454 | 4.1700 | **<0.0001** |
| **Intermediate Concentration** | 0.0646 | 0.0454 | 1.4200 | 0.1500 |
| **High Concentration** | 0.0028 | 0.0467 | 0.0600 | 0.9500 |
| **Log(scale)** | -1.1798 | 0.0414 | -28.4900 | **<0.0001** |
|  | **exp^(β)^** | **se(β)** | ***z-value*** | **P-value** |
| **(ii)MMCoxPH** |  |  |  |  |
| **Survival 5 hpl** |  |  |  |  |
| **Low Concentration** | 0.4330 | 0.4578 | -1.8300 | 0.0680 |
| **Intermediate Concentration** | 0.5033 | 0.4378 | -1.5700 | 0.1200 |
| **High Concentration** | 0.7277 | 0.4083 | -0.7800 | 0.4400 |

Results evaluating (i) the effect of CFSE different concentrations on cercarial survival after 24 hpl with regression Weibull model (RWM) (alive cercariae ~ CFSE concentration) and (ii) after 5 hpl with mixed model Cox Proportional Hazards regression (MMCoxPH) (alive cercariae ~ CFSE concentrations + replicates (random)). The hazard rate (exp^(β)^) of control cercariae in (ii) is 1, to which the other levels are compared. If exp^(β)^ < 1, mortality risk is reduced. Statistically significant results (at α = 0.050) are indicated in bold. We also provide the scale parameter which indicates with log(scale) the Weibull distribution estimation.
