# Supplementary material for: In vivo fluorescent cercariae reveal the entry portals of Cardiocephaloides longicollis (Rudolphi, 1819) Dubois, 1982 (Strigeidae) into the gilthead seabream Sparus aurata L
Source: Parasit Vectors. 2019 Mar 12;12:92. doi: 10.1186/s13071-019-3351-9 (PMC6417200; doi:10.1186/s13071-019-3351-9)
Supplement: Supplementary file 5 — Additional file 5: Table S5. Evaluation of the effect of CFSE concentration treatment on cercarial activity. [file 13071_2019_3351_MOESM5_ESM.docx]

**Additional file 5: Table S5**. Evaluation of the effect of CFSE concentration treatment on cercarial activity.

Increase in activity of cercariae labelled with low and intermediate CFSE concentration after 5 hpl.

|  | **Estimate** | **SE** | ***z-value*** | **P-value** |
| --- | --- | --- | --- | --- |
| **RWM** |  |  |  |  |
| **Activity 5 hpl** |  |  |  |  |
| **Intercept (=Control)** | 2.4802 | 0.1292 | 19.2000 | **<0.0001** |
| **Low Concentration** | 0.8622 | 0.2284 | 3.7700 | **0.0002** |
| **Intermediate Concentration** | 0.4019 | 0.1976 | 2.0300 | **0.0420** |
| **High Concentration** | 0.1065 | 0.1856 | 0.5700 | 0.5660 |
| **Log(scale)** | -0.2269 | 0.0827 | -2.7400 | **0.0061** |

Results of regression Weibull model (RWM) evaluating the effect of CFSE concentration treatment on cercariae activity rate at 5 hpl (active cercariae ~ CFSE concentration). The intercept value stands for the activity rate of the control, to which the other three levels are compared, i.e. low, intermediate and high CFSE concentration. Statistically significant results (at α = 0.050) are indicated in bold. We also provide the scale parameter which indicates with log(scale) the Weibull distribution estimation.
