# Supplementary material for: In vivo fluorescent cercariae reveal the entry portals of Cardiocephaloides longicollis (Rudolphi, 1819) Dubois, 1982 (Strigeidae) into the gilthead seabream Sparus aurata L
Source: Parasit Vectors. 2019 Mar 12;12:92. doi: 10.1186/s13071-019-3351-9 (PMC6417200; doi:10.1186/s13071-019-3351-9)
Supplement: Supplementary file 6 — Additional file 6: Table S6. Evaluation of the effect of dyes on cercarial survival and activity. [file 13071_2019_3351_MOESM6_ESM.docx]

**Additional file 6: Table S6**. Evaluation of the effect of dyes on cercarial survival and activity.

Increase of survival and activity of cercariae labelled with NB intermediate concentration after 5 hpl. Increase in activity of cercariae labelled with CFSE after 5 hpl.

|  | **Estimate** | **SE** | ***z-value*** | **P-value** |
| --- | --- | --- | --- | --- |
| **RWM** |  |  |  |  |
| **(i) Survival** |  |  |  |  |
| **Intercept (=Control)** | 4.3180 | 0.4820 | 8.9600 | **<0.0001** |
| **NB** | 1.8100 | 0.6620 | 2.7300 | **0.0062** |
| **CFSE** | 0.9020 | 0.5360 | 1.6800 | 0.0925 |
| **Log(scale)** | 0.1660 | 0.1810 | 0.9200 | 0.3588 |
| **(ii) Activity** |  |  |  |  |
| **Intercept (=Control)** | 2.4741 | 0.1288 | 19.2000 | **<0.0001** |
| **NB** | 0.6641 | 0.1935 | 3.4300 | **0.0006** |
| **CFSE** | 0.3964 | 0.1958 | 2.0200 | **0.0429** |
| **Log(scale)** | -0.2398 | 0.0917 | -2.6200 | **0.0089** |

Results of regression Weibull model (RWM) evaluating the effect of NB and CFSE intermediate concentration on (i) cercarial survival (alive cercariae ~ treatment) and (ii) activity rates (active cercariae ~ treatment) after 5 hpl. The intercept value in (i) stands for the survival rate and in (ii) for the activity rate, of the control cercariae, which the other two levels are compared, i.e. NB and CFSE both with intermediate concentration. Statistically significant results (at α = 0.050) are indicated in bold. We also provide the scale parameter which indicates with log(scale) the Weibull distribution estimation.
