# Supplementary material for: In vivo fluorescent cercariae reveal the entry portals of Cardiocephaloides longicollis (Rudolphi, 1819) Dubois, 1982 (Strigeidae) into the gilthead seabream Sparus aurata L
Source: Parasit Vectors. 2019 Mar 12;12:92. doi: 10.1186/s13071-019-3351-9 (PMC6417200; doi:10.1186/s13071-019-3351-9)
Supplement: Supplementary file 7 — Additional file 7: Table S7. Evaluation of the effect of major regions of fish’s surface on cercarial density. [file 13071_2019_3351_MOESM7_ESM.docx]

**Additional file 7: Table S7**. Evaluation of the effect of major regions of fish’s surface on cercarial density.

Higher density of attached cercariae on the fish’s head compared to the fins.

|  | **Estimate** | **SE** | ***t-value*** | **P-value** |
| --- | --- | --- | --- | --- |
| **(i) LMM** |  |  |  |  |
| **Intercept (=Head)** | 2.8160 | 0.1554 | 18.1190 | **<0.0001** |
| **Body** | 0.4223 | 0.2198 | 1.9210 | 0.0556 |
| **Fin** | 0.6809 | 0.1966 | 3.4640 | **0.0006** |
|  | **Estimate** | **SE** | ***z-value*** | **P-value** |
| **(ii) Pairwise comparison** |  |  |  |  |
| **Body – Head** | 0.4223 | 0.2198 | 1.9210 | 0.1321(0.1641) |
| **Fin – Head** | 0.6809 | 0.1966 | 3.4640 | **0.0015(0.0016)** |
| **Fin – Body** | 0.2586 | 0.1966 | 1.3160 | 0.3854(0.5649) |

Results of (i) linear mixed model (LMM) (attached cercariae density ~ major region + replicates (random)) and (ii) pairwise comparison evaluating the effect of major regions on cercarial density, calculated as number of cercariae/area cm^2^ (Box-Cox transformed values). The intercept value in the LMM stands for the mean density of cercariae attached to the head region, to which the other two regions are compared, i.e. body and fins. The estimate of each variable is added to the intercept value. Statistically significant results (at α = 0.050) are indicated in bold, with the corresponding P-value obtained after Bonferroni correction given in parentheses. We also provide: random effect ‘replicates’, variance < 0.001.
