# Supplementary material for: In vivo fluorescent cercariae reveal the entry portals of Cardiocephaloides longicollis (Rudolphi, 1819) Dubois, 1982 (Strigeidae) into the gilthead seabream Sparus aurata L
Source: Parasit Vectors. 2019 Mar 12;12:92. doi: 10.1186/s13071-019-3351-9 (PMC6417200; doi:10.1186/s13071-019-3351-9)
Supplement: Supplementary file 8 — Additional file 8: Table S8. Evaluation of the effect of sub-regions of fish’s surface on cercarial density. [file 13071_2019_3351_MOESM8_ESM.docx]

**Additional file 8: Table S8**. Evaluation of the effect of sub-regions of fish’s surface on cercarial density.

Higher density of attached cercariae on the eyes, gills, lower side body and dorsal fin compared to the rest of sub-regions.

|  | | **Estimate** | **SE** | ***t-value*** | **P-value** |
| --- | --- | --- | --- | --- | --- |
| **(i) LMM** | |  |  |  |  |
| **Intercept (=H1, Eye)** | 0.9644 | | 0.0900 | 10.7160 | **<0.0001** |
| **H2, Mouth** | 0.3558 | | 0.1258 | 2.8280 | **0.0050** |
| **H3, Gills** | 0.1408 | | 0.1258 | 1.1200 | 0.2638 |
| **B1, Upper side** | 0.3014 | | 0.1258 | 2.3960 | **0.0172** |
| **B2, Middle side** | 0.2842 | | 0.1258 | 2.2590 | **0.0246** |
| **B3, Lower side** | 0.1363 | | 0.1258 | 1.0840 | 0.2794 |
| **F1 Dorsal fin** | 0.0655 | | 0.1258 | 0.5210 | 0.6030 |
| **F2, Pectoral fin** | 0.3679 | | 0.1258 | 2.9250 | **0.0037** |
| **F3, Pelvic fin** | 0.3814 | | 0.1258 | 3.0320 | **0.0026** |
| **F4, Anal fin** | 0.3575 | | 0.1258 | 2.8400 | **0.0048** |
| **F5, Caudal fin** | 0.6169 | | 0.1258 | 4.9030 | **<0.0001** |
|  | **Estimate** | | **SE** | ***z-value*** | **P-value** |
| **(ii) Pairwise comparison** |  | |  |  |  |
| **Mouth – Eye** | 0.3558 | | 0.1258 | 2.8280 | 0.1469(0.2575) |
| **Gills – Eye** | 0.1408 | | 0.1258 | 1.1200 | 0.9898(1.0000) |
| **Upper side – Eye** | 0.3014 | | 0.1258 | 2.4000 | 0.3692(0.9122) |
| **Middle side – Eye** | 0.2842 | | 0.1258 | 2.2590 | 0.4626(1.0000) |
| **Lower side – Eye** | 0.1363 | | 0.1258 | 1.0840 | 0.9921(1.0000) |
| **Dorsal fin – Eye** | 0.0655 | | 0.1258 | 0.5200 | 0.9999(1.0000) |
| **Pectoral fin – Eye** | 0.3679 | | 0.1258 | 2.9250 | 0.1153(0.1897) |
| **Pelvic fin – Eye** | 0.3814 | | 0.1258 | 3.0320 | 0.0866(0.1337) |
| **Anal fin – Eye** | 0.3575 | | 0.1258 | 2.8420 | 0.1417(0.2466) |
| **Caudal fin – Eye** | 0.6169 | | 0.1258 | 4.9030 | **<0.0001(<0.0001)** |
| **Gills – Mouth** | -0.2150 | | 0.1258 | -1.7090 | 0.8314(1.0000) |
| **Upper side – Mouth** | -0.0544 | | 0.1258 | -0.4320 | 1.0000(1.0000) |
| **Middle side – Mouth** | -0.0715 | | 0.1258 | -0.5700 | 1.0000(1.0000) |
| **Lower side – Mouth** | -0.2195 | | 0.1258 | -1.7440 | 0.8124(1.0000) |
| **Dorsal fin – Mouth** | -0.2903 | | 0.1258 | -2.3070 | 0.4287(1.0000) |
| **Pectoral fin – Mouth** | 0.0121 | | 0.1258 | 0.0960 | 1.0000(1.0000) |
| **Pelvic fin – Mouth** | 0.0256 | | 0.1258 | 0.2040 | 1.0000(1.0000) |
| **Anal fin – Mouth** | 0.0017 | | 0.1258 | 0.0140 | 1.0000(1.0000) |
| **Caudal fin – Mouth** | 0.2611 | | 0.1258 | 2.0800 | 0.5954(1.0000) |
| **Upper side – Gills** | 0.1606 | | 0.1258 | 1.2760 | 0.9728(1.0000) |
| **Middle side – Gills** | 0.1434 | | 0.1258 | 1.1400 | 0.9882(1.0000) |
| **Lower side – Gills** | -0.0045 | | 0.1258 | -0.0360 | 1.0000(1.0000) |
| **Dorsal fin – Gills** | -0.0753 | | 0.1258 | -0.5990 | 1.0000(1.0000) |
| **Pectoral fin – Gills** | 0.2271 | | 0.1258 | 1.8050 | 0.7775(1.0000) |
| **Pelvic fin – Gills** | 0.2406 | | 0.1258 | 1.9120 | 0.7090(1.0000) |
| **Anal fin – Gills** | 0.2167 | | 0.1258 | 1.7220 | 0.8242(1.0000) |
| **Caudal fin – Gills** | 0.4760 | | 0.1258 | 3.7840 | **0.0075(0.0085)** |
| **Middle side – Upper side** | -0.0172 | | 0.1258 | -0.1360 | 1.0000(1.0000) |
| **Lower side – Upper side** | -0.1651 | | 0.1258 | -1.3120 | 0.9670(1.0000) |
| **Dorsal fin – Upper side** | -0.2359 | | 0.1258 | -1.8750 | 0.7337(1.0000) |
| **Pectoral fin – Upper side** | 0.0665 | | 0.1258 | 0.5290 | 1.0000(1.0000) |
| **Pelvic fin – Upper side** | 0.0800 | | 0.1258 | 0.6360 | 0.9999(1.0000) |
| **Anal fin – Upper side** | 0.0561 | | 0.1258 | 0.4460 | 1.0000(1.0000) |
| **Caudal fin – Upper side** | 0.3155 | | 0.1258 | 2.5080 | 0.2993(0.6686) |
| **Lower side – Middle side** | -0.1479 | | 0.1258 | -1.1760 | 0.9851(1.0000) |
| **Dorsal fin – Middle side** | -0.2187 | | 0.1258 | -1.7390 | 0.8153(1.0000) |
| **Pectoral fin – Middle side** | 0.0837 | | 0.1258 | 0.6650 | 0.9999(1.0000) |
| **Pelvic fin – Middle side** | 0.0972 | | 0.1258 | 0.7720 | 0.9995(1.0000) |
| **Anal fin – Middle side** | 0.0733 | | 0.1258 | 0.5830 | 1.0000(1.0000) |
| **Caudal fin – Middle side** | 0.3326 | | 0.1258 | 2.6440 | 0.2263(0.4508) |
| **Dorsal fin – Lower side** | -0.0708 | | 0.1258 | -0.5630 | 1.0000(1.0000) |
| **Pectoral fin – Lower side** | 0.2316 | | 0.1258 | 1.8410 | 0.7561(1.0000) |
| **Pelvic fin – Lower side** | 0.2451 | | 0.1258 | 1.9480 | 0.6853(1.0000) |
| **Anal fin – Lower side** | 0.2212 | | 0.1258 | 1.7580 | 0.8046(1.0000) |
| **Caudal fin – Lower side** | 0.4805 | | 0.1258 | 3.8200 | **0.0065(0.0074)** |
| **Pectoral fin – Dorsal fin** | 0.3024 | | 0.1258 | 2.4040 | 0.3633(0.8922) |
| **Pelvic fin – Dorsal fin** | 0.3160 | | 0.1258 | 2.5110 | 0.2972(0.6618) |
| **Anal fin – Dorsal fin** | 0.2920 | | 0.1258 | 2.3210 | 0.4176(1.0000) |
| **Caudal fin – Dorsal fin** | 0.5514 | | 0.1258 | 4.3830 | **0.0006(0.0006)** |
| **Pelvic fin – Pectoral fin** | 0.0135 | | 0.1258 | 0.1070 | 1.0000(1.0000) |
| **Anal fin – Pectoral fin** | -0.0104 | | 0.1258 | -0.0830 | 1.0000(1.0000) |
| **Caudal fin – Pectoral fin** | 0.2489 | | 0.1258 | 1.9790 | 0.6637(1.0000) |
| **Anal fin – Pelvic fin** | -0.0239 | | 0.1258 | -0.1900 | 1.0000(1.0000) |
| **Caudal fin – Pelvic fin** | 0.2354 | | 0.1258 | 1.8710 | 0.7364(1.0000) |
| **Caudal fin – Anal fin** | 0.2593 | | 0.1258 | 2.0610 | 0.6051(1.0000) |
|  |  | |  |  |  |

Results of (i) linear mixed model (LMM) (attached cercariae density ~ sub-region + replicates (random))) and (ii) pairwise comparison evaluating the effect of sub-regions on cercarial density, calculated as number of cercariae/area cm^2^ (Box-Cox transformed values). Fish surface is divided in 11 regions, representing the original fish division and so, subdividing the head into 3 sub-regions (eye (H1), mouth (H2), gills (H3)), the body into 3 sub-regions (upper (B1), middle (B2) and lower (B3) side), and the fins into 5 sub-regions (dorsal (F1) pectoral (F2), pelvic (F3), anal (F4) and caudal (F5) fins). The intercept value in the LMM stands for the mean density of cercariae attached to the fish eye sub-region (H1), to which the other sub-regions are compared. The estimate of a variable is added to the intercept value. Statistically significant results (at α = 0.050) are indicated in bold, with the corresponding P-value obtained after Bonferroni correction given in parentheses. We also provide: random effect ‘replicates’, variance = 0.003.
