# Supplementary material for: In vivo fluorescent cercariae reveal the entry portals of Cardiocephaloides longicollis (Rudolphi, 1819) Dubois, 1982 (Strigeidae) into the gilthead seabream Sparus aurata L
Source: Parasit Vectors. 2019 Mar 12;12:92. doi: 10.1186/s13071-019-3351-9 (PMC6417200; doi:10.1186/s13071-019-3351-9)
Supplement: Supplementary file 9 — Additional file 9: Table S9. Evaluation of the effect of dyes on cercarial infectivity and metacercarial encystment success. [file 13071_2019_3351_MOESM9_ESM.docx]

**Additional file 9: Table S9**. Evaluation of the effect of dyes on cercarial infectivity and metacercarial encystment success.

Lower infection rate of cercariae labelled with CFSE compared to control cercariae.

|  | **Estimate** | **SE** | ***z-value*** | **P-value** |
| --- | --- | --- | --- | --- |
| **(i)GLMM** |  |  |  |  |
| **Intercept (=Control)** | -1.8811 | 0.4713 | -3.9930 | **<0.0001** |
| **NB** | -0.2240 | 0.1743 | -1.2850 | 0.1988 |
| **CFSE** | -0.5363 | 0.1855 | -2.8910 | **0.0039** |
| **(ii) Pairwise comparison** |  |  |  |  |
| **Control – NB** | 0.2240 | 0.1743 | 1.2850 | 0.4032(0.5963) |
| **CFSE – NB** | -0.3123 | 0.1914 | -1.6310 | 0.2320(0.3084) |
| **CFSE – Control** | -0.5363 | 0.1855 | -2.8910 | **0.0105(0.0115)** |

Results of (i) generalized linear mixed model (GLMM) (proportion of brain-encysted metacercariae ~ dye type + replicates (random)) and (ii) pairwise comparison evaluating the effect of NB and CFSE on cercarial infectivity and metacercariae encystment success. The intercept value in the GLMM stands for the mean number of cercariae control, encysted as metacercariae in the fish’s brain on the logit scale, to which the other treatments are compared. The estimate of a variable is added or subtracted to the intercept value. Statistically significant results (at α = 0.050) are indicated in bold, with the corresponding P-value obtained after Bonferroni correction given in parentheses. We also provide random effect ‘replicates’, variance = 0.616.
