# Supplementary material for: In vivo fluorescent cercariae reveal the entry portals of Cardiocephaloides longicollis (Rudolphi, 1819) Dubois, 1982 (Strigeidae) into the gilthead seabream Sparus aurata L
Source: Parasit Vectors. 2019 Mar 12;12:92. doi: 10.1186/s13071-019-3351-9 (PMC6417200; doi:10.1186/s13071-019-3351-9)
Supplement: Supplementary file 10 — Additional file 10: Table S10. Evaluation of the effect of fish’s head sub-regions on cercarial density. [file 13071_2019_3351_MOESM10_ESM.docx]

**Additional file 10: Table S10**. Evaluation of the effect of fish’s head sub-regions on cercarial density.

Lower density of attached cercariae on gills compared to the rest of fish’s head sub-regions.

|  | **Estimate** | **SE** | ***t-value*** | **P-value** |
| --- | --- | --- | --- | --- |
| **(i) LMM** |  |  |  |  |
| **Intercept (=H1, Eye)** | 0.9677 | 0.0091 | 105.8990 | **<0.0001** |
| **H2, Mouth** | 0.0480 | 0.0129 | 3.6990 | **0.0004** |
| **H3, Gills** | 0.0159 | 0.0129 | 1.2280 | 0.2230 |
|  | **Estimate** | **SE** | ***z-value*** | **P-value** |
| **(ii) Pairwise comparison** |  |  |  |  |
| **Mouth – Eye** | 0.0478 | 0.0129 | 3.6990 | **<0.0001(0.0001)** |
| **Gills – Eye** | 0.0159 | 0.0129 | 1.2280 | 0.4365(0.4360) |
| **Gills – Mouth** | -0.0319 | 0.0129 | -2.4700 | **0.0361(0.0360)** |

Results of (i) linear mixed model (LMM) (attached cercariae density of only fish head surface ~only head sub-regions + replicates (random)) and (ii) pairwise comparison evaluating the effect of head sub-regions on cercarial density, calculated as number of cercariae/area cm^2^ (Box-Cox transformed values). The fish head surface is divided into 3 sub-regions (eye (H1), mouth (H2), gills (H3)). The intercept value in the LMM stands for the mean density of cercariae attached to the fish eye sub-region (H1), to which the other head sub-regions are compared. The estimate of a variable is added to the intercept value. Statistically significant results (at α = 0.050) are indicated in bold, with the corresponding P-value obtained after Bonferroni correction given in parentheses. We also provide random effect ‘replicates’, variance <0.001.
